# Knowledge, attitudes, and practices toward cardiovascular complications among end-stage renal disease patients undergoing maintenance hemodialysis

**DOI:** 10.1186/s12889-024-18945-5

**Published:** 2024-05-30

**Authors:** Zhuo Li, Li Song, Ruifang Hua, Fangxiao Xia, Duanfeng Hu, Zhenghui Luo, Jianteng Xie, Sijia Li, Zhonglin Feng, Shuangxin Liu, Jianchao Ma, Ting Lin, Renwei Huang, Feng Wen, Lei Fu, Sheng Li, Hao Dai, Dongmei Cui, Qizhen Liang, Xiaoli Kang, Minfen Liu, Zhiming Ye

**Affiliations:** 1 Department of Nephrology, Guangdong Provincial People’s Hospital, Guangdong Academy of Medical Sciences, Guangzhou, 510080 PR China; 2 Department of Nephrology, Ganzhou Hospital of Guangdong Provincial People’s Hospital, Ganzhou Municipal Hospital, Ganzhou, 341099 China; 3 Department of Nephrology, Shangyou People’s Hospital, Ganzhou, 341299 China

**Keywords:** Knowledge, attitudes, practices, Renal Dialysis, Cardiovascular diseases, Web-based study

## Abstract

**Background:**

This study aimed to investigate the knowledge, attitudes, and practices (KAP) toward cardiovascular complications among end-stage renal disease patients undergoing maintenance hemodialysis.

**Methods:**

This web-based cross-sectional study was conducted at Guangdong Provincial People’s Hospital between December 2022, and May 2023.

**Results:**

A total of 545 valid questionnaires were collected, with an average age of 57.72 ± 13.47 years. The mean knowledge, attitudes and practices scores were 8.17 ± 2.9 (possible range: 0–24), 37.63 ± 3.80 (possible range: 10–50), 33.07 ± 6.10 (possible range: 10–50) respectively. Multivariate logistic regression analysis showed that patients from non-urban area had lower knowledge compared to those from urban area (odds ratio (OR) = 0.411, 95% CI: 0.262–0.644, *P* < 0.001). Furthermore, higher levels of education were associated with better knowledge, as indicated by OR for college and above (OR = 4.858, 95% CI: 2.483–9.504), high school/vocational school (OR = 3.457, 95% CI: 1.930–6.192), junior high school (OR = 3.300, 95% CI: 1.945–5.598), with primary school and below as reference group (all *P* < 0.001). Besides, better knowledge (OR = 1.220, 95% CI: 1.132–1.316, *P* < 0.001) and higher educational levels were independently associated with positive attitudes. Specifically, individuals with a college degree and above (OR = 2.986, 95% CI: 1.411–6.321, *P* = 0.004) and those with high school/vocational school education (OR = 2.418, 95% CI: 1.314–4.451, *P* = 0.005) have more positive attitude, with primary school and below as reference group. Next, better attitude (OR = 1.174, 95% CI: 1.107–1.246, *P* < 0.001) and higher education were independently associated with proactive practices. Those with college and above (OR = 2.870, 95% CI: 1.359–6.059, *P* = 0.006), and those with high school/vocational school education (OR = 1.886, 95% CI: 1.032–3.447, *P* = 0.039) had more proactive practices, with primary school and below as reference group.

**Conclusions:**

End-stage renal disease patients undergoing maintenance hemodialysis demonstrated insufficient knowledge, positive attitudes, and moderate practices regarding cardiovascular complications. Targeted interventions should prioritize improving knowledge and attitudes, particularly among patients with lower educational levels and income, to enhance the management of cardiovascular complications in end-stage renal disease.

## Background

End-stage renal disease is a widespread health problem affecting millions of individuals worldwide. Approximately 4 million patients worldwide are currently receiving kidney replacement therapy (KRT), with hemodialysis being the most prevalent form, constituting approximately 69% of all KRT and 89% of all dialysis procedures [1]. However, the rates of impaired quality of life, morbidity, and mortality among hemodialysis patients are alarmingly high [2]. Notably, cardiovascular complications emerge as a major concern. It was reported that cardiovascular disease affects more than two-thirds of individuals undergoing hemodialysis and stands as the leading cause of morbidity, contributing to nearly 50% of all mortalities in this population [1]. The high prevalence of cardiovascular disease among patients undergoing hemodialysis is likely due to various factors, including fluid and electrolyte imbalances, hemodynamic instability, and the presence of uremic toxins during the dialysis process [3–5]. These factors can exert significant strain on the cardiovascular system, contributing to the increased risk of cardiovascular complications in these patients. Managing these risks requires a comprehensive approach to optimize fluid balance, control comorbid conditions, and closely monitor cardiovascular health in hemodialysis patients [6]. The coexistence of end-stage renal disease and cardiovascular disease creates a complex clinical scenario, necessitating a comprehensive understanding of the interplay between these two conditions.

In the quest to improve the care and outcomes of maintenance hemodialysis patients, investigating their knowledge, attitudes, and practices (KAP) regarding cardiovascular complications is of paramount importance. The KAP framework provides valuable insights into how patients perceive and manage their health conditions, offering a comprehensive assessment of their awareness, attitudes towards risk reduction, and adherence to recommended practices [7]. By exploring the KAP of maintenance hemodialysis patients, healthcare providers can identify gaps in patient education and implement targeted interventions to address modifiable risk factors and enhance cardiovascular health.

Despite the wealth of research on cardiovascular complications in patients with end-stage renal disease undergoing hemodialysis, there is a critical gap in understanding how these patients perceive and manage their cardiovascular health. A study conducted in Finland revealed that there was a significant lack of knowledge concerning dialysis management among patients undergoing pre-dialysis or home dialysis [8]. Previous studies have highlighted the significant improvement with the knowledge-attitude-behavior education program for Chinese adults undergoing maintenance hemodialysis [9]. However, there remains a paucity of research focusing specifically on the KAP of maintenance hemodialysis patients regarding cardiovascular complications. Therefore, the primary objective of this study is to explore the KAP toward cardiovascular complications among end-stage renal disease patients undergoing maintenance hemodialysis.

## Methods

### Study design and patients

This cross-sectional study was conducted at the Blood Purification Center of Guangdong Provincial People’s Hospital between December 2022, and May 2023. The study enrolled maintenance hemodialysis patients with end-stage renal disease who met the following inclusion criteria: (1) End-stage renal disease patients receiving maintenance hemodialysis for three months or more; (2) Age ≥ 18 years; and (3) Signed informed consent form. Patients who were unable to independently complete the survey were excluded from the study.

This study was approved by the Ethics Committee of Guangdong Provincial People’s Hospital (KY-Q-2022-435-01) and obtained informed consent from the study subjects before completing the questionnaire.

### Questionnaire and quality control

According to the Chinese Guidelines for the Management of Chronic Kidney Disease during Peri-dialysis Period [10], Chinese Guidelines for the Management of Chronic Heart Failure in Dialysis Patients [11], and the Clinical practice guidelines for renal anemia in China [12], a self-administered questionnaire consisting of four dimensions was developed and reviewed by three experts, including two nephrology specialists and one statistician, and incorporated for improvements. Similar questions were eliminated, and adjustments were made to clarify ambiguous statements. The final questionnaire consists of four sections: (1) Demographic characteristics include 8 items. (2) Knowledge dimension consists of 12 questions. A score of 2 signifies a high level of understanding, 1 is assigned for recognition (heard of), and 0 is allotted for incorrect or unclear answers. (3) Attitude dimension includes 10 questions. A five-point Likert scale was used, ranging from “very positive” (5 points) to “very negative” (1 point). (4) Practice dimension involves 10 questions, with the same five-point Likert scale, ranging from “always” (5 points) to “never” (1 point).

A small-scale pilot study involving 80 patients was conducted. The internal consistency of the questionnaire was evaluated good internal consistency with Cronbach’s α was 0.865. For data collection, an online questionnaire was developed using the Wen Juan Xing (WJX) platform (http s://www.wjx.cn), and a quick response (QR) code was generated for patients to access the questionnaire through WeChat. To ensure data quality and completeness, measures were taken to limit each IP address to a single submission, and all fields were made mandatory. Furthermore, the research team members meticulously examined the questionnaires for completeness, internal consistency, and reasonableness.

### Statistical analysis

The statistical analysis software used was Stata 17.0 (Stata Corporation, College Station, TX, USA). Quantitative variables were described using mean ± standard deviation (SD), and between-group comparisons were conducted using t-tests or analysis of variance (ANOVA). Categorical variables were described using n (%) proportions. Pearson correlation analysis was used to assess the correlation between knowledge, attitude, and practice scores. Logistic regression analysis was employed for univariate and multivariate analyses of knowledge, attitudes and practices, with a cut-off value of 70% based on the score distribution [13]. In the multiple regression model, variables with a univariate P-value < 0.05 were included. Two-sided P-value < 0.05 was considered significantly different.

## Results

### Demographic characteristic

A total of 622 questionnaires were received. Subsequently, 66 of these were excluded from the analysis due to missing or abnormal values in the basic information, and 11 more questionnaires were excluded due to missing or abnormal KAP responses. As a result, the final analysis included 545 valid questionnaires. Among the patients, the average age was 57.72 ± 13.47 years, and the average dialysis duration was 5.00 ± 5.18 years. Among them, 306 (56.15%) were male, and 334 (61.28%) resided in urban areas. Moreover, 227 (41.65%) had an educational level of primary school and below, while only 54 (9.91%) were employed. Additionally, 287 (52.66%) reported a monthly per capita income < 2,000 CNY. Patients residing in urban areas and possessing higher education levels were more likely to demonstrate markedly higher knowledge (all *P* < 0.001), attitude (all *P* < 0.001), and practice (all *P* < 0.001) scores (Table 1).

**Table 1:** Baseline characteristics and KAP scores

Variables	*N* (%)	Knowledge Score		Attitude Score		Practice Score	
		Mean ± SD	*P*	Mean ± SD	*P*	Mean ± SD	*P*
**Total Score**	545	8.17±2.90		37.63±3.80		33.07±6.10	
**Gender**			0.128		0.886		0.362
Male	306(56.15)	8.34±2.81		37.65±3.76		32.86±6.09	
Female	239(43.85)	7.96±3.01		37.61±0.3.86		33.34±6.11	
**Age**	57.72±13.47						
**Residence**			< 0.001		< 0.001		< 0.001
Urban	334(61.28)	8.78±2.92		38.39±3.96		33.97±6.00	
Non-urban	211(38.72)	7.21±2.60		36.44±3.19		31.65±6.00	
**Education**			< 0.001		< 0.001		< 0.001
Primary school and below	227(41.65)	7.03±2.70		36.10±3.04		31.37±5.79	
Junior high school	128(23.49)	8.61±3.03		37.84±3.93		33.22±6.47	
High school/vocational school	109(20.00)	9.16±2.70		38.99±3.58		34.60±5.46	
College and above	81(14.86)	9.36±2.36		39.78±4.02		35.57±5.82	
**Employment**			0.020		< 0.001		< 0.001
Employed	54(9.91)	8.41±2.68		38.93±4.05		33.74±6.28	
Unemployed	162(29.72)	7.81±2.53		37.06±3.79		32.01±6.61	
Retired	157(28.81)	8.73±3.03		38.61±3.95		35.00±6.07	
Other	172(31.56)	7.94±3.12		36.87±3.28		32.10±5.08	
**Monthly Per Capita Income, CNY**			0.002		< 0.001		0.003
< 2000	287(52.66)	7.83±2.89		36.91±3.32		32.24±5.69	
2000–5000	166(30.46)	8.29±2.96		37.98±4.27		33.86±6.53	
> 5000	92(16.88)	9.03±2.66		39.26±3.76		34.26±6.21	
Dialysis Duration/years	5.00±5.18			-		-	
**Underlying Diseases (Multiple Choice)**							
Diabetes	183(33.58)			-		-	
Hypertension	460(84.40)			-		-	
Chronic nephritis	118(21.65)			-		-	
Other	35(6.42)			-		-	
**Health Insurance (Multiple Choice)**							
Urban Employee Basic Medical Insurance	219(40.18)			-		-	
New Rural Cooperative Medical Insurance	71(13.03)			-		-	
Urban Resident Basic Medical Insurance	244(44.77)			-		-	
Retired Cadre Medical Insurance	6(1.10)			-		-	
Commercial Insurance	3(0.55)			-		-	
No Insurance	6(1.10)			-		-	

The average knowledge, attitude and practice scores were 8.17 ± 2.9 (possible range: 0–24), 37.63 ± 3.80 (possible range: 10–50), 33.07 ± 6.10 (possible range: 10–50) respectively (Table 1). The distribution of knowledge indicated that patients did not attain a satisfactory level of knowledge concerning cardiovascular complications. The three knowledge items with the highest correctness rates were as follows: *“Anemia significantly increases the risk of cardiovascular events and death in patients, severely affecting quality of life and survival rate.”* with a correctness rate of 81.10%; *“Anemia promotes the progression of chronic kidney disease and is significantly associated with heart failure in dialysis patients”*, with a correctness rate of 79.45%; and *“Advanced age, smoking, alcohol consumption, obesity, and diabetes are all risk factors for cardiovascular events such as heart failure”*, with a correctness rate of 75.83%. However, the item with the lowest correctness rate was *“The blood pressure control target for diabetic kidney disease patients on dialysis is < 140/90 mmHg”*, with a correctness rate of 35.41%. The attitude distribution revealed that the majority of patients displayed a predominantly positive attitude towards cardiovascular complications, with “Agree” being the most commonly chosen response. Specifically, 34.68% of patients strongly agreed with the statement *“I believe that preventing and treating the occurrence and progression of cardiovascular events is beneficial for prolonging life and improving quality of life”.* Conversely, only 3.49% of patients strongly disagreed with the statement *“If suspected of heart failure, I think following the doctor’s instructions for various tests is too cumbersome”.* For the practices, most patients reported *“Quitting smoking”* (61.83%) and *“Taking antihypertensive medication as prescribed by the doctor”* (58.9%) as their primary actions. However, only 20.37% of patients chose “Always” in the statement “Adjusting diet, sodium intake of 5-6 g per day”, and a notable 22.39% indicated “Never” for the practice of “Quitting smoking.” (Table 2).

**Table 2:** Knowledge, attitudes, and practices

Items, *n* (%)							
Knowledge					Accuracy		
**1. The incidence of cardiovascular events (heart failure, arrhythmia, etc.) is high in dialysis patients and is currently the leading cause of death.**					390(71.56)		
**2. The main manifestations of heart failure are difficulty breathing, fatigue, and fluid retention.**					434(79.63)		
**3. The prevalence of heart failure is high in patients with chronic kidney disease, and the incidence of heart failure increases with worsening kidney function.**					374(68.62)		
**4. Diabetes and hypertension do not affect the risk of cardiovascular events. (False)**					351(64.40)		
**5. Heart failure and end-stage renal disease often mutually influence and promote each other, leading to further deterioration of prognosis.**					386(70.83)		
**6. Hypertension is the most common complication in hemodialysis patients, so regular monitoring is sufficient and no intervention is needed. (False)**					356(65.32)		
**7.Anemia significantly increases the risk of cardiovascular events and death in patients, severely affecting quality of life and survival rate.**					442(81.10)		
**8. The blood pressure control target for diabetic kidney disease patients on dialysis is < 140/90 mmHg. (False)**					193(35.41)		
**9. Advanced age, smoking, alcohol consumption, obesity, and diabetes are all risk factors for cardiovascular events such as heart failure**					428(75.83)		
**10. Anemia promotes the progression of chronic kidney disease and is significantly associated with heart failure in dialysis patients.**					433(79.45)		
**11. Mineral and bone abnormalities do not increase the risk of heart failure. (False)**					258(47.34)		
**12. To control interdialytic weight gain, daily salt intake should be < 5 g.**					409(75.05)		
**Attitude**		**Strongly Agree**	**Agree**	**Neutral**		**Disagree**	**Strongly Disagree**
**1. I feel anxious about the occurrence of cardiovascular complications. (N)**		87(15.96)	234(42.94)	149(27.34)		70(12.84)	5(0.92)
**2. I am willing to learn more about cardiovascular complications related to end-stage renal disease and hemodialysis. (P)**		175(32.11)	306(56.15)	45(8.26)		15(2.75)	4(0.73)
**3. I believe that preventing and treating the occurrence and progression of cardiovascular events is beneficial for prolonging life and improving quality of life. (P)**		189(34.68)	310(56.88)	37(6.79)		5(0.92)	4(0.73)
**4. I think I should strengthen the monitoring of symptoms and signs related to heart failure. (P)**		126(23.12)	317(58.17)	89(16.33)		12(2.20)	1(0.18)
**5. If suspected of heart failure, I think following the doctor’s instructions for various tests is too cumbersome. (N)**		46(8.44)	207(37.89)	112(20.55)		161(29.54)	19(3.49)
**6. I believe that blood pressure changes should be closely monitored and antihypertensive medications should be taken as prescribed. (P)**		163(29.911)	327(60.00)	52(9.54)		2(0.37)	1(0.18)
**7. I believe that interdialytic weight gain should be controlled. (P)**		121(22.20)	351(64.40)	62(11.38)		10(1.83)	1(0.18)
**8. I believe that we should be vigilant about anemia and take corrective measures such as taking iron supplements when necessary. (P)**		156(28.62)	329(60.37)	51(9.36)		7(1.28)	2(0.37)
**9. I believe that managing blood sugar and blood lipids is very important. (P)**		132(24.22)	275(50.46)	133(24.40)		2(0.37)	3(0.55)
**10. If I cannot tolerate intermittent hemodialysis, I am willing to follow medical advice and adopt a continuous renal replacement therapy plan. (P)**		75(13.76)	260(47.71)	141(25.87)		62(11.38)	7(1.28)
**Practice**	**Always**		**Often**	**Sometimes**		**Occasionally**	**Never**
**1. Adjusting diet, sodium intake of 5–6 g per day (P)**	111(20.37)		196(35.96)	143(26.24)		80(14.68)	15(2.75)
**2. Limiting water intake as advised by the doctor (P)**	160(29.36)		203(37.25)	101(18.53)		68(12.48)	13(2.39)
**3.Quitting smoking (P)**	337(61.83)		44(8.07)	24(4.40)		18(3.30)	122(22.39)
**4.Limiting alcohol consumption (P)**	309(56.70)		96(17.61)	35(6.42)		14(2.57)	91(16.70)
**5. Controlling weight, avoiding excessive weight loss or obesity, weight gain between dialysis sessions < 4% (P)**	123(22.57)		229(42.02)	132(24.22)		30(5.50)	31(5.69)
**6 Regular exercise (P)**	90(16.51)		133(24.40)	117(21.47)		87(15.96)	118(21.65)
**7. Adding fiber-rich foods to the diet (P)**	90(16.51)		237(43.49)	156(28.62)		51(9.36)	11(2.02)
**8. Managing blood sugar well (P)**	148(27.16)		148(27.16)	88(16.15)		55(10.09)	106(19.45)
**9. Regular follow-up, monitoring relevant indicators (P)**	261(47.89)		211(38.72)	51(9.36)		16(2.94)	6(1.10)
**10. Taking antihypertensive medication as prescribed by the doctor (P)**	321(58.90)		177(32.48)	21(3.85)		6(1.10)	20(3.67)

Pearson correlation analysis showed that knowledge was positively associated with attitude (*r* = 0.414, *P* < 0.001) and practice (*r* = 0.213, *P* < 0.001). Moreover, the analysis revealed that attitudes was positively associated with practice (*r* = 0.399, *P* < 0.001) (Table 3).

**Table 3:** Pearson correlation analysis

	Knowledge	Attitude	Practice
Knowledge	1		
Attitude	0.414 (*P* < 0.001)	1	
Practice	0.213 (*P* < 0.001)	0.399 (*P* < 0.001)	1

Multivariate logistic regression analysis showed that patients from non-urban area had lower knowledge compared to those from urban area (odds ratio (OR) = 0.411, 95% CI: 0.262–0.644, *P* < 0.001). Furthermore, higher levels of education were associated with better knowledge, as indicated by OR for college and above (OR = 4.858, 95% CI: 2.483–9.504), high school/vocational school (OR = 3.457, 95% CI: 1.930–6.192), junior high school (OR = 3.300, 95% CI: 1.945–5.598), with primary school and below as reference group (all *P* < 0.001). Besides, better knowledge (OR = 1.220, 95% CI: 1.132–1.316, *P* < 0.001) and higher educational levels were independently associated with positive attitudes. Specifically, individuals with a college degree and above (OR = 2.986, 95% CI: 1.411–6.321, *P* = 0.004) and those with high school/vocational school education (OR = 2.418, 95% CI: 1.314–4.451, *P* = 0.005) have more positive attitude, with primary school and below as reference group. Next, better attitude (OR = 1.174, 95% CI: 1.107–1.246, *P* < 0.001) and higher education were independently associated with proactive practices. Those with college and above (OR = 2.870, 95% CI: 1.359–6.059, *P* = 0.006), and those with high school/vocational school education (OR = 1.886, 95% CI: 1.032–3.447, *P* = 0.039) had more proactive practices, with primary school and below as reference group (Table 4).

**Table 4:** Univariate and multivariate logistic regression analysis

	Variables	Univariate logistic regression		Multivariate logistic regression	
		OR (95%CI)	*P*	OR (95%CI)	*P*
**Knowledge**	**Gender**				
	Male	ref			
	Female	0.884(0.621–1.257)	0.492		
	**Age**	0.995(0.983–1.008)	0.493		
	**Residence**				
	Urban	Ref		ref	
	Non-urban	0.272(0.182–0.407)	< 0.001	0.411(0.262–0.644)	< 0.001
	**Education Level**				
	Primary school and below	ref		Ref	
	Junior high school	3.759(2.314–6.107)	< 0.001	3.300(1.945–5.598)	< 0.001
	High school/vocational school	4.139(2.496–6.862)	< 0.001	3.457(1.930–6.192)	< 0.001
	College and above	6.271(3.597–10.934)	< 0.001	4.858(2.483–9.504)	< 0.001
	**Employment Status**				
	Employed	ref			
	Unemployed	0.634(0.330–1.217)	0.170		
	Retired	1.516(0.803–2.860)	0.199		
	Other	0.911(0.483–1.719)	0.773		
	**Monthly Per Capita Income, yuan**				
	< 2000	0.723(0.484–1.080)	0.113	1.547(0.956–2.503)	0.075
	2000–5000	ref		ref	
	> 5000	1.708(1.020–2.858)	0.042	1.250(0.715–2.185)	0.433
	**Dialysis Duration, years**	1.042(1.007–1.079)	0.019	1.022(0.986–1.058)	0.240
**Attitude**	**Knowledge**	1.289(1.203–1.381)	< 0.001	1.220(1.132–1.316)	< 0.001
	**Gender**				
	Male	ref			
	Female	0.936(0.666–1.315)	0.704		
	**Age**	0.983(0.971–0.996)	0.009	0.988(0.972–1.005)	0.182
	**Residence**				
	Urban	ref		ref	
	Non-urban	0.416(0.290–0.595)	< 0.001	0.933(0.593–1.469)	0.765
	**Education Level**				
	Primary school and below	ref		ref	
	Junior high school	2.760(1.749–4.355)	< 0.001	1.662(0.982–2.811)	0.058
	High school/vocational school	4.723(2.899–7.694)	< 0.001	2.418(1.314–4.451)	0.005
	College and above	7.636(4.308–13.536)	< 0.001	2.986(1.411–6.321)	0.004
	**Employment Status**				
	Employed	ref		ref	
	Unemployed	0.358(0.187–0.689)	0.002	0.896(0.402–1.999)	0.789
	Retired	0.586(0.304–1.128)	0.110	0.824(0.368–1.845)	0.638
	Other	0.205(0.106–0.396)	< 0.001	0.545(0.238–1.245)	0.150
	**Monthly Per Capita Income, yuan**				
	< 2000	0.551(0.374–0.813)	0.003	0.902(0.539–1.508)	0.693
	2000–5000	ref		ref	
	> 5000	2.286(1.334–3.916)	0.003	1.678(0.926–3.043)	< 0.088
	**Dialysis Duration, years**	1.019(0.986–1.054)	0.252		
**Practice**	**Knowledge**	1.148(1.079–1.222)	< 0.001	1.026(0.954–1.104)	0.946
	**Attitude**	1.222(1.159–1.289)	< 0.001	1.174(1.107–1.246)	< 0.001
	**Gender**				
	Male	ref			
	Female	1.038(0.739–1.458)	0.829		
	**Age**	1.004(0.992–1.017)	0.497		
	**Residence**				
	Urban	ref		ref	
	Non-urban	0.584(0.441–0.831)	0.003	1.327(0.846–2.080)	0.218
	**Education Level**				
	Primary school and below	ref		ref	
	Junior high school	2.243(1.432–3.514)	< 0.001	1.586(0.950–2.648)	0.078
	High school/vocational school	3.396(2.110–5.468)	< 0.001	1.886(1.032–3.447)	0.039
	College and above	5.052(2.924–8.727)	< 0.001	2.870(1.359–6.059)	0.006
	**Employment Status**				
	Employed	ref		ref	
	Unemployed	0.497(0.266–0.928)	0.028	0.864(0.399–1.871)	0.711
	Retired	1.137(0.607–2.128)	0.689	1.450(0.719–2.925)	0.299
	Other	0.368(0.197–0.688)	0.002	0.714(0.324–1.5730	0.404
	**Monthly Per Capita Income, yuan**				
	< 2000	0.495(0.335–0.729)	< 0.001	0.940(0.568–1.557)	0.811
	2000–5000	ref		ref	
	> 5000	1.098(0.657–1.834)	0.722	0.715(0.399–1.283)	0.261
	**Dialysis Duration, years**	1.012(0.980–1.046)	0.474		

## Discussion

The study revealed that end-stage renal disease patients undergoing maintenance hemodialysis demonstrated inadequate knowledge, positive attitudes, and moderate practices toward cardiovascular complications. These findings underscore the importance of improving attitudes and providing educational support to encourage more proactive practices in managing cardiovascular risks among this patient group. Targeted interventions should prioritize enhancing knowledge and attitudes, particularly among patients with lower education levels and lower income, to enhance the management of cardiovascular complications in this population.

The findings from our study indicated that the average scores for KAP were generally below optimal levels, suggesting a widespread lack of adequate knowledge and appropriate practices concerning cardiovascular complications in patients with chronic kidney disease (CKD). Although the KAP of cardiovascular complications in patients with CKD or on hemodialysis was not established, it was reported that poor knowledge about CKD among the public and patients with CKD was widely reported in Iran [14], Hong Kong [15], and Northern Tanzania [16]. For example, a study conducted in Nigeria revealed that 65% of the patients with CKD exhibited poor knowledge about their condition [17]. Notably, research has demonstrated that patients’ knowledge is closely linked to improved self-management behaviors in patients on hemodialysis [18]. This study further showed a positive correlation between knowledge and practice scores, emphasizing the importance of enhancing the knowledge of patients on hemodialysis, particularly regarding cardiovascular complications. It is crucial to address this knowledge gap, as most therapies aimed at preventing kidney disease progression and reducing associated complications heavily rely on patients’ ability to effectively self-manage their condition.

Socioeconomic factors, such as residing in urban areas, higher education levels, and greater monthly per capita income, were associated with higher KAP scores. These findings suggest a socioeconomic disparity in cardiovascular health awareness and practices among maintenance hemodialysis patients. A study in United Kingdom reported that low socioeconomic status is related to severity of CKD [19]. Similarly, a study among US adults also showed that lower socioeconomic status including limited education or lower income is associated with greater risk of disability from CKD [20]. Moreover, a study of 2,171 patients with peritoneal dialysis in China showed that low personal income independently predicted the highest risks for all-cause or cardiovascular death compared with medium and high income [21]. Furthermore, the multivariate logistic regression analysis in this study identified higher attitude scores and higher education levels as independent predictors of more proactive practices. This finding highlights the significance of education in empowering individuals to engage in self-management behaviors to prevent the cardiovascular complications in their condition. Consistently, it was reported that the tertiary educational level was the only significant independent predictor of higher CKD knowledge (OR = 2.62 95%CI: 1.20–5.72, *P* = 0.02) [17]. It is likely due to the fact that individuals with more resources and access to educational opportunities are better equipped to the knowledge of measures and engage more effectively in preventing cardiovascular complications. Therefore, healthcare systems should focus on implementing targeted interventions that address the specific needs of patients from lower socioeconomic backgrounds.

In the context of preventing cardiovascular complications on maintenance hemodialysis, lifestyle modifications play a pivotal role, encompassing actions such as restricting dietary salt and water intake, engaging in regular exercise, and discontinuing smoking [22]. However, the current study reveals that patients with end-stage renal disease undergoing hemodialysis demonstrated only moderate adherence to practices aimed at mitigating cardiovascular complications. Specifically, a relatively modest portion of patients (20.37%) reported consistently adjusting their dietary habits to maintain a recommended sodium intake. Moreover, the cardiovascular care provided to hemodialysis patients should not only address their dietary salt but also ensure the promotion of good nutrition for those in the end-stage renal disease category. This dual focus is vital as cardiovascular complications may be interconnected with the malnutrition-inflammation-atherosclerosis syndrome in chronic kidney disease [23]. Nevertheless, a notable subset (22.39%) still expressed resistance to the practice of quitting smoking. It was widely established that smoking is associated with a markedly increased risk of heart disease in dialysis patients [24, 25]. Indeed, the findings of this study underscore the critical significance of lifestyle interventions for patients undergoing dialysis to effectively prevent cardiovascular complications. Besides education to the patients, it was also important for the doctors to maintaining good health practice upon themselves, and share experience to patients.

It is noteworthy that most participants in this study, who were undergoing maintenance hemodialysis for end-stage renal disease, were predominantly elderly, with lower educational attainment and reduced household income. This demographic profile correlated with demonstrated insufficient knowledge, positive attitudes, and moderate practices concerning cardiovascular complications. Given these findings, it is imperative to provide practical and easily understandable guidelines tailored to their unique circumstances. This could encompass straightforward dietary recommendations, simple exercise routines, and stress management techniques adapted to the lifestyle and resources of this population. Additionally, the establishment of community-based support groups is recommended. These groups can offer a platform for participants to share experiences, discuss challenges, and celebrate successes related to their health. Such forums serve as a valuable source of encouragement, fostering a sense of community and collective well-being among individuals facing similar health journeys.

The present study has several limitations. One limitation of this study was its cross-sectional design, which precludes the establishment of causal relationships between KAP scores and other factors. Longitudinal studies with interventions targeted at improving knowledge and attitudes are needed to further validate our findings and assess their impact on patients’ practices and cardiovascular outcomes over time. Certain questions may be deemed advanced for CKD patients, this is particularly evident due to the evolving nature of clinical practice guidelines and the ongoing debates among elite clinicians on topics such as blood pressure control targets and the role of anemia in CKD progression. However, it is crucial to acknowledge that participants’ responses to these questions may be influenced by their attentiveness to health education provided by medical personnel. Therefore, these questions can still be considered a valuable gauge of patients’ engagement with health education, providing insights into their understanding. Moreover, some terms (e.g., arrhythmias, heart failure, fatigue, etc.) used in the knowledge and attitude-related items could be considered technical for some patients. To ensure a clear understanding of the terms used in the questionnaire, oral explanations were provided to participants.

## Conclusion

In conclusion, end-stage renal disease patients undergoing maintenance hemodialysis demonstrated inadequate knowledge, positive attitudes, and moderate practices related to cardiovascular complications. To enhance the management of cardiovascular complications in this patient group, it is crucial to implement targeted interventions aimed at improving knowledge and attitudes, particularly among those with lower education levels and lower income. By addressing these factors, better outcomes in the management of cardiovascular complications may be achieved among individuals undergoing hemodialysis for end-stage renal disease.

## Data Availability

All data generated or analysed during this study are included in this published article.

## Abbreviations

KRT: Kidney replacement therapy
KAP: Knowledge, attitudes, and practices
WJX: Wen Juan Xing
QR: Quick response
SD: Standard deviation
ANOVA: Analysis of variance
CKD: Chronic kidney disease
